# Supplementary material for: Macroevolutionary brain scaling is a microevolutionary metaphenomenon
Source: Nat Commun. 2025 Dec 4;17:136. doi: 10.1038/s41467-025-66843-0 (PMC12775500; doi:10.1038/s41467-025-66843-0)
Supplement: Supplementary file 1 — Supplementary Infomation [file 41467_2025_66843_MOESM1_ESM.pdf]

Supplementary Information for:

Macroevolutionary brain scaling is a microevolutionary metaphenomenon

Authors: Joanna Baker[1]*, Robert A. Barton[2], and Chris Venditti[1]*

Affiliations: [1]School of Biological Sciences, University of Reading; Reading, RG6 6BX, UK

[2]Department of Anthropology, Durham University; South Road, Durham, DH1 3LE, UK

*__Corresponding authors__

Email: j.l.a.baker@reading.ac.uk; c.d.venditti@reading.ac.uk

This PDF file includes:

Supplementary Note 1: Subgroups identified as different from global curvature

Supplementary Note 2: Significance of curvature

Supplementary Note 3: Summary of rate heterogeneity

Supplementary Note 4: Bias in slope parameters without non-significant clades

Supplementary Note 5: Results using a single-rate model of evolution

Supplementary Note 6: Theoretical expectations derived from within-species allometry

Supplementary Note 7: Body size dependency in within-species allometry

Supplementary Note 8: The effect of common brain size covariates on size-dependency

Supplementary Note 9: Analyses incorporating uncorrected within-species variation

Supplementary Figs. S1 to S14

Supplementary Tables S1 to S11

Supplementary References

Supplementary Text

Supplementary Note 1: Subgroups identified as different from global curvature

In our main analysis, we find a significant curved relationship between brain and body size across all animal species. Using deviation contrast coding we find that no single class of animal differs from a global curvature estimated across all species (all $p_x > 0.05$, **Fig. 1, inset**). However, when we divide each of the major animal groups into their constituent subclades (see **Materials & Methods**), we identify five animal subclades that differ from a global curvature estimated across all species (**Fig. 2A**): Atlantogenata (mammals), Charadriformes and Falconiformes (birds), Urodela (amphibians), and Labriformes (fish).

For each subclade, we investigated why we may have observed deviations. To do this, we identified potential outliers as well as split each subclade into two or more monophyletic groups (henceforth *sub-subclades*) based on phylogenetic relationships and data variability. We then studied each sub-subclades in a modified version of our subclade curve model (henceforth referred to as our suite of *sub-subclade curve models*) limited only to the class for which each of the original subclades belonged and removing potential outliers. Not only is this computationally more efficient, but we have already demonstrated (in our class curve models) that no individual class differs significantly from the global curvature estimated across all species. As in our main text, the major animal clades are indicated by coloured silhouettes.

Urodela

Urodela is an order of amphibians containing all extant salamanders. Urodela shows significantly negative curvature (median quadratic coefficient = -0.16, $p_x = 0.012$) that is significantly greater in magnitude (i.e., more negative) than that observed across all other taxa ($p_{x[diff]} = 0.047$, see **Fig. 2A**) – although this difference is marginal and does not reach significance in all replicates. When we repeat our subclade curve model at the amphibian level, our conclusions are identical: There is significant negative curvature (median quadratic coefficient = -0.18, $p_x = 0.002$) and a strongly significant difference from the amphibian curve ($p_{x[diff]} = 0.014$). However, it should be noted that owing to the way the grand mean is calculated, both subclades (Anura and Urodela) – whilst tending towards negative values – significantly differ from the grand mean, as they fall either side.

Most salamanders in our dataset fall within the suborder Salamandroidea (N = 52), with only N = 3 species falling into the Cryptobranchoidea suborder. Cryptobranchoidea is too small for further study and so was excluded from all further sub-subclade analyses. There are two major subclades within Salamandroidea that each form a monophyletic group in the tree (**Fig. S1**) and contain several named families (clade 1: N = 26, Ambystomatidae, Dicampodontidae,

Salamandridae; clade 2: N = 26, Rhyacotritonidae, Amphiumidae, Plethodontidae, and Proteidae). Both sub-subclades overlap in variation although there is considerably more variability observed in clade 2 (**Fig. S1**). There are two species (both in clade 2) with very tiny body size – pygmy salamanders belonging to the genus *Thorius* (**Fig. S1**). Additionally, there are two species (one from each of the two clades) that have a seemingly much larger brain size than expected given the variation observed in all other species. In our outlier analyses, we tested a model that removed all four of these taxa.

When we divide Urodela into the smaller clades, we find that *no* amphibian group (including Anura) differs from the global amphibian curvature. Both clades tend towards negative curvature (median quadratic coefficient = -0.142 and -0.256 for clade 1 and 2 respectively). However, clade 1 is poorly estimated, spanning a wide range of parameter values whereas clade 2 retains a clearly steeper (i.e. more negative) quadratic parameter (median quadratic coefficient = -0.256) – although it is still non-significant with respect to the amphibian curvature (p_x = 0.256, **Fig. S1**). Anurans still tend towards negative curvature (median quadratic coefficient for Anura = -0.017). The result is qualitatively identical after the exclusion of the potential outliers.

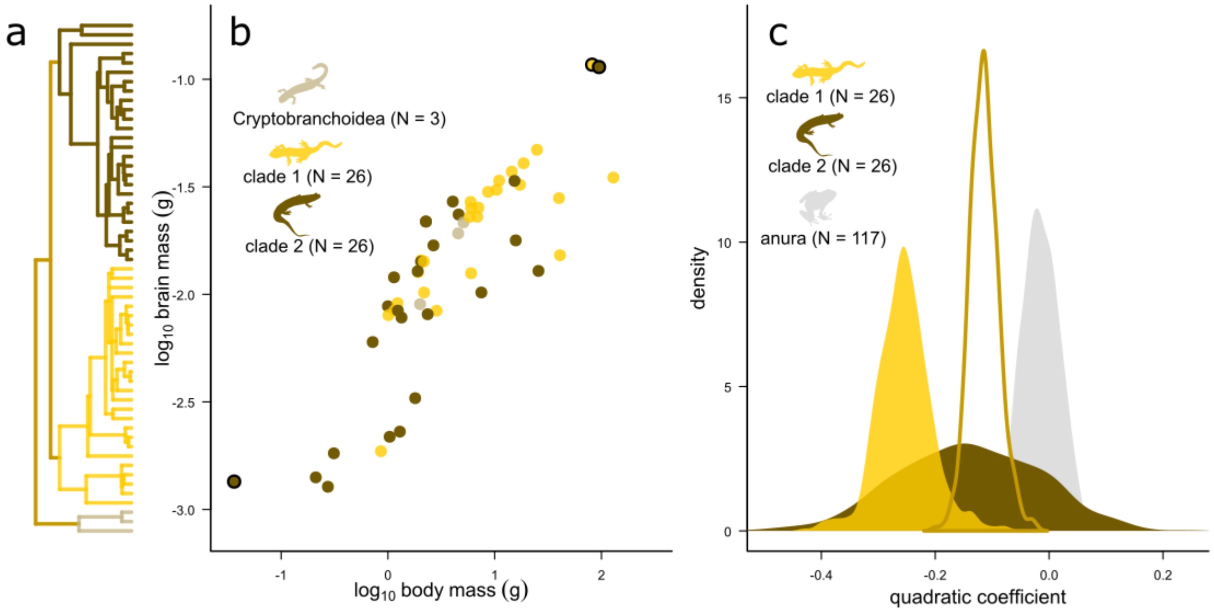

Fig. S1 Exploring the curvature within Urodela. *(a) The Urodela phylogeny with branches coloured by assignment to three major monophyletic sub-clades. (b) Brain and body size data for Urodela, coloured by sub-clade. (c) Difference in estimated quadratic parameter for each of the two sub-clades large enough for further study as well as for anurans (grey) compared to the grand mean curvature estimated across all amphibians (solid gold outline distribution). None of the three sub-clade distributions significantly deviates from the amphibian-level quadratic.*

Labriformes

Labriformes is an order of ray-finned fishes including the parrotfishes and wrasses. This subclade demonstrates significantly negative curvature (median quadratic coefficient = -0.06, p_x = 0.000) that is significantly greater in magnitude (i.e., more negative) than that observed across all other taxa ($p_{x[diff]}$ = 0.043, see **Fig. 2A**). When we limit our analysis to fish, our conclusions are similar; though not identical. Whilst we do observe a curvature of similar magnitude (median quadratic coefficient = -0.06, p_x = 0.000), this is not substantially significantly different from the grand mean curvature estimated across all fish ($p_{x[diff]}$ = 0.108).

Whilst the curvature across fish substantially overlaps that observed across all animals (**Fig. 1, inset**), the global mean estimated across fish in isolation is slightly lower (median quadratic coefficient = -0.04). In our full analysis, Labriformes was only marginally significantly different from the global curvature estimated across all other animals ($p_{x[diff]}$ = 0.043) and in some replicates this value was non-significant. It is therefore likely that this is only a marginal difference. In any case, we continued to explore the variation in Labriformes compared to other fish.

Labriformes can broadly be separated into three major groups that also form distinct monophyletic clades on the phylogenetic tree (**Fig. S2**): hogfish (N = 6), parrotfish (N = 40, note that here we refer to this clade as parrotfish but that this does also contains a few species of wrasse) and wrasses (N = 49). There are too few species of hogfish for further study and so these are excluded from all further sub-subclade analyses. We therefore opted to study parrotfish and wrasses as two separate sub-subclades.

Within wrasses, there are two possible outliers: the belted wrasse (*Stethojulis balteata*) is much smaller both in brain and body size than any other species belonging to this order, and the clown coris (*Coris aygula*) has a very tiny brain for its body size compared to other wrasses (**Fig. S2**). We therefore removed both taxa from our outlier analyses.

When we study parrotfish and wrasses separately, neither clade differs from a global curvature estimated across all fish (median quadratic coefficient = -0.041, p_x = 0.434 and median quadratic coefficient = -0.068, p_x = 0.068 for parrotfish and wrasses respectively, see **Fig. S2**). The result is indistinguishable after exclusion of the potential outliers. While there may be some marginal effect in this group leading to a slightly steeper curve than other animals, the qualitative pattern remains the same: a negative quadratic relationship.

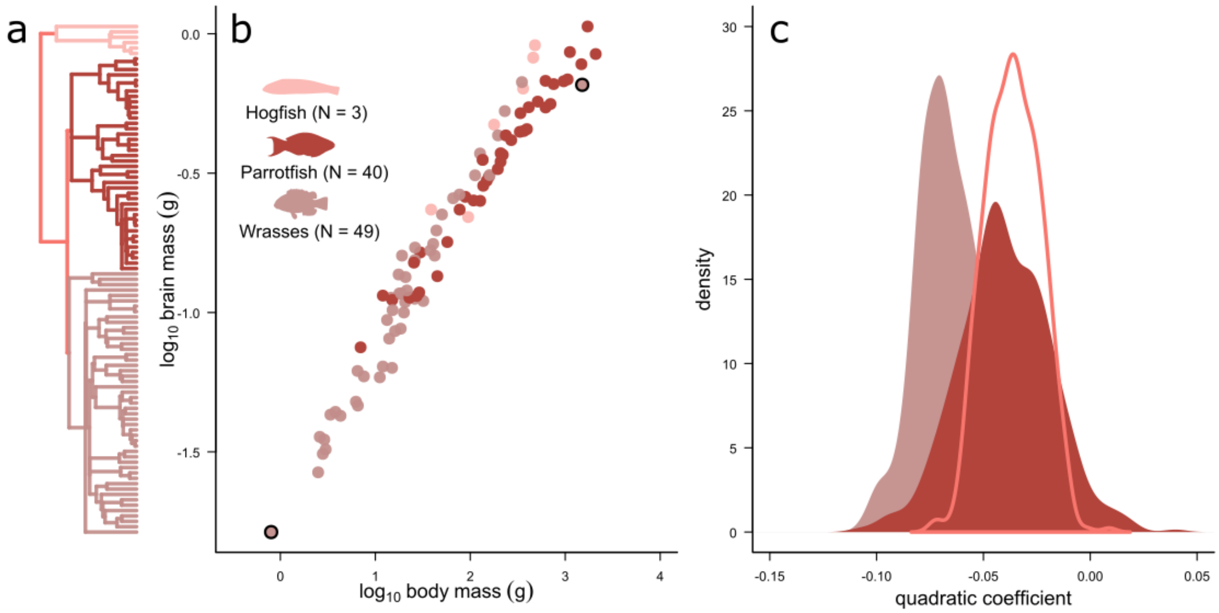

Fig. S2 Exploring the curvature within Labriformes. *(a) The Labriformes phylogeny, with branches coloured by assignment to three major sub-clades, hogfish, parrotfish, and wrasses. (b) Brain and body size data for Labriformes, coloured by sub-clade. (c) Difference in estimated quadratic parameter for each of the two sub-clades large enough for further study compared to the grand mean curvature estimated across all fish (solid coral outline distribution). Neither distribution significantly deviates from the fish-level quadratic.*

Falconiformes

Falconiformes is an order of carnivorous birds of prey including falcons and caracaras with negative curvature (median quadratic coefficient = -0.23, p_x = 0.007) that is significantly greater in magnitude (i.e., more negative) than observed across all other taxa ($p_{x[diff]}$ = 0.009, see **Fig. 3A**). When we limit our analysis to only include birds, our conclusions are identical (median quadratic coefficient = -0.18, p_x = 0.008, $p_{x[diff]}$ = 0.008).

There is no clear way to separate Falconiformes into monophyletic clades, although there are two small-bodied species (falconets and pygmy falcons) that are possible outliers **(Fig. S3B)**. When these two species are removed from the analysis, the curvature for Falconiformes is highly similar (median quadratic coefficient = -0.23) but is no longer statistically distinct from the global bird curve ($p_{x[diff]}$ = 0.07) – the estimate spans a much wider range of potential curvatures (**Fig. S3C**). It is possible that these two tiny Falconiformes are simply much smaller – both in terms of their brains, and body sizes – than would be expected given the variation observed in the rest of the order.

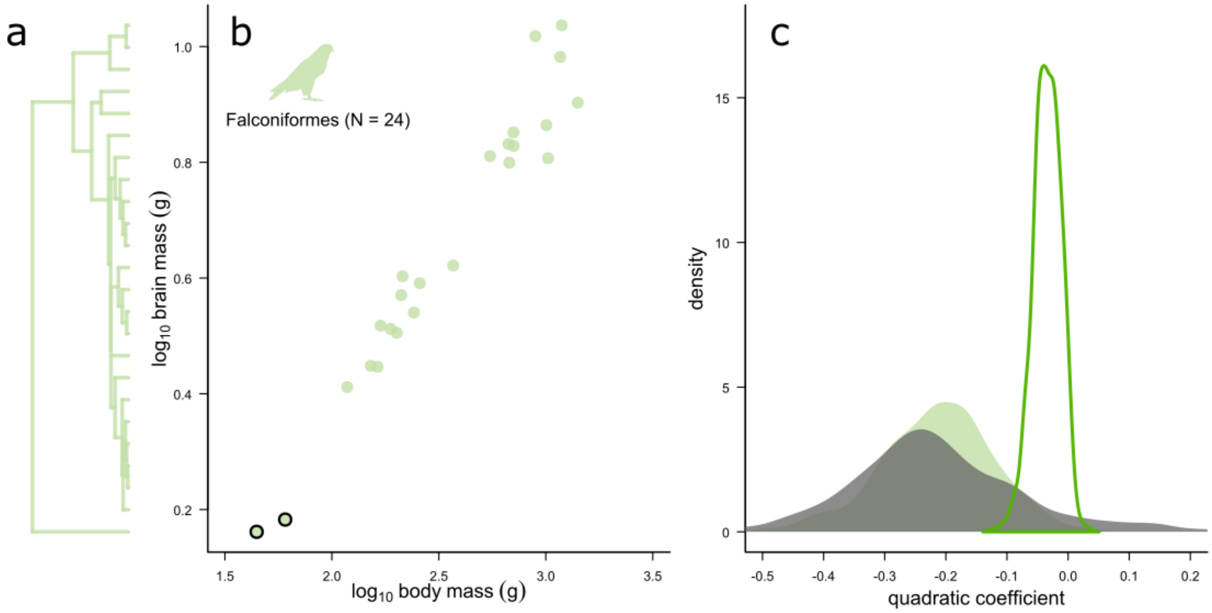

Fig. S3 Exploring the curvature within Falconiformes. *(a) Falconiformes phylogeny. (b) Brain and body size data, with potential outliers outlined in black. (c) Difference in estimated quadratic parameter for all species (green) and excluding potential outliers (grey) compared to the grand mean curvature estimated across all birds (solid green outline distribution).*

Atlantogenata

Atlantogenata is a magnorder of mammals (N = 47) that includes both the order Xenarthra (N = 15, sloths and armadillos) and the superorder Afrotheria (N = 32, which is itself comprised of multiple orders of African origin, including elephants, sirenians, elephant shrews, and the aardvark). The curvature in this subclade is positive (median quadratic coefficient = 0.012, p_x = 0.01) and is significantly different to that observed across all other animals ($p_{x[diff]}$ = 0.001, where $p_{x[diff]}$ is the proportion of the posterior distribution of differences between the subclade-level quadratic parameter and the global quadratic parameter). When we limit our analysis to mammals, our conclusions are similar: there is an overall positive curvature (median quadratic coefficient = 0.05, p_x = 0.01) that significantly differs from the mammal curve ($p_{x[diff]}$ = 0.002).

Xenarthra and Afrotheria are quite distinct, having diverged ~ 96 million years ago (**Fig. S4A, inset).** Xenarthra (N = 15) spans only a moderate range of brain and body size whereas Afrotheria (N = 32) exhibits enormous variation (**Fig. S4B**). We thus opted to study Xenartha and Afrotheria as separate sub-subclades. Whilst Xenarthra (N = 15) falls below our threshold sample size criteria for inclusion in our main analysis, we include it here for comparative purposes. Notably, elephants (N = 2) and dugongs and manatees (N = 3) fall well outside the range of variation observed by all other groups (**Fig. S4B**) and were tested as potential outliers.

We constructed a version of our subclade curve model that estimated a separate quadratic effect for each mammalian subclade, dividing Afrotheria and Xenarthra. In this mammalian sub-subclade curve model, Xenarthra exhibits a generally negative curve (median quadratic coefficient = -0.16) that does not significantly differ from a global mammalian curvature ($p_{x[diff]}$ = 0.07, **Fig. S4C**). In contrast, Afrotheria is positive (median quadratic coefficient = 0.06) and remains significantly different from other mammals($p_{x[diff]}$ = 0.000, **Fig. S4C**). However, when the model is repeated excluding Sirenia (manatees and dugongs, N = 3) and Proboscidea (elephants, N = 2), this effect disappears. That is, after the exclusion of these large-bodied orders, Afrotheria no longer significantly differs to the global curvature estimated across all species ($p_{x[diff]}$ = 0.128, **Fig. S4C**). Why these species may fundamentally differ from all other mammalian species is an unexplored, yet important, question.

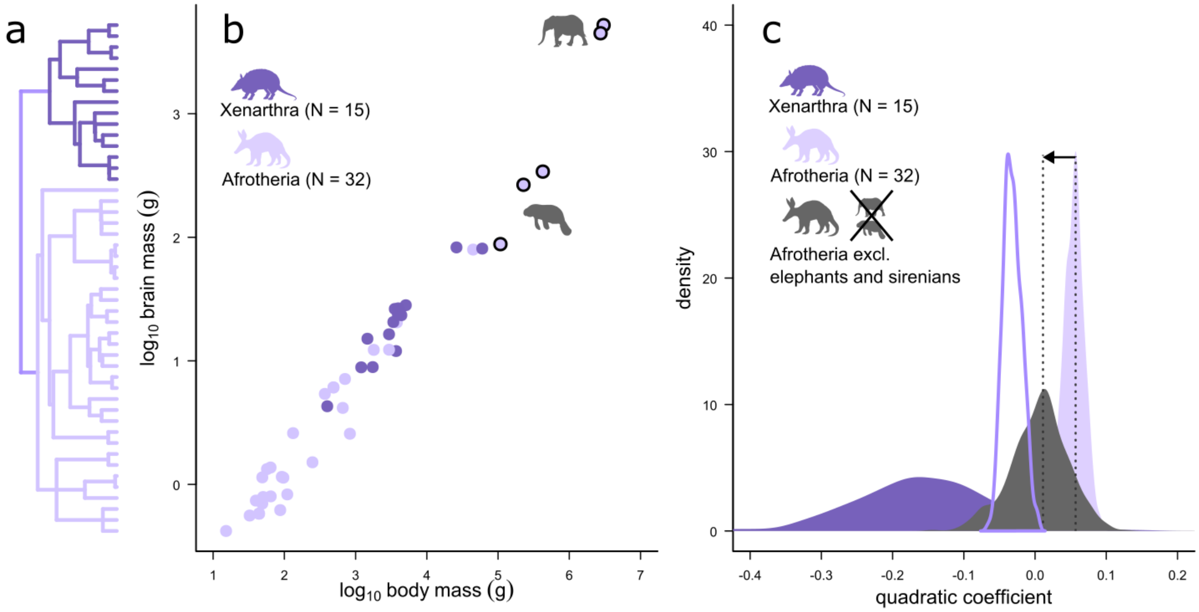

Fig. S4 Exploring the curvature within Atlantogenata. *(a) Phylogenetic tree with branches coloured by their assignment to the two major sub-clades, Xenarthra and Afrotheria. (b) The brain and body size data for all taxa coloured by their assignment to sub-clade and with potential outliers outlined in black. (c) The difference in estimated quadratic parameter for Xenarthra and Afrotheria compared to the grand mean curvature estimated across all mammals (purple outlined distribution). There are two distributions for Afrotheria: the first, in light purple, shows the estimated relationship across all taxa, whereas the second, in dark grey, shows the estimated relationship excluding the large-bodied sirenians and elephants. Note that removing these few species shifts the distribution (median values indicated by the dotted vertical lines) such that the distribution no longer diverges from the grand mean curvature.*

Charadriiformes

Charadriiformes (N = 140) is a large group of shorebirds. The curvature in this subclade is positive (median quadratic coefficient = 0.06, p_x = 0.010) and differs from the global curvature observed across all other animals ($p_{x[diff]}$ = 0.000). When we limit our analysis to birds, our conclusions are identical: There is significant positive curvature (median quadratic coefficient = 0.06, p_x = 0.011) and a strongly significant difference from the global bird curve ($p_{x[diff]}$ = 0.000).

Charadriiformes is comprised of three clearly distinct sub-subclades which fall within a basal polytomy (**Fig. S5**). These broadly correspond to the three suborders of the order: Charadrii (N = 35), Scolopaci (N = 47), and Lari (N = 58). We therefore split the subclade into these three suborders for further study. There are no obvious potential outliers (**Fig. S5**, and so we did not remove any members of this order from our outlier analyses.

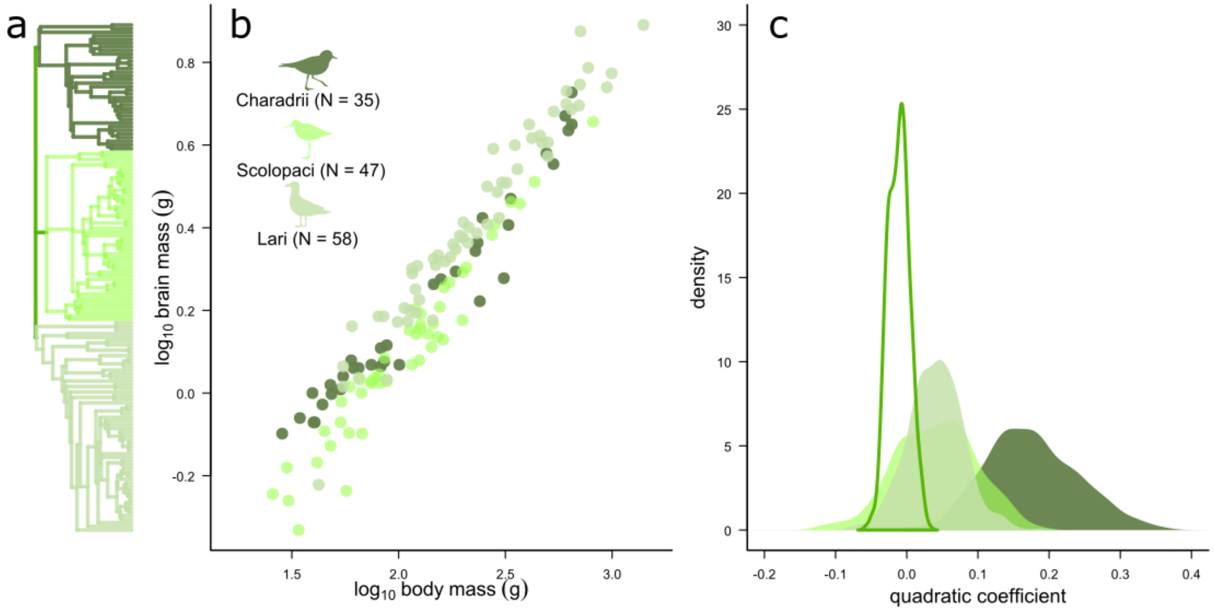

Fig. S5 Exploring the curvature within Charadriiformes. *(a) The phylogenetic structure of the group is shown, with branches coloured by assignment to three major sub-clades, Charadrii, Scolopaci, and Lari. (b) Brain and body size data for Charadriiformes, coloured by sub-clade. (c) Difference in estimated quadratic parameter for each of the three sub-clades compared to the grand mean curvature estimated across all birds (solid green outline distribution). Only the distribution for Charadrii (darkest green) significantly deviates from the bird-level quadratic.*

Within shorebirds, it seems as though the only group with significant positive curvature is Charadrii (median quadratic coefficient = 0.17 p_x = 0.002). While the other two groups also display positive curvature (median quadratic coefficients = 0.043 and 0.042 for Scolopaci and

Lari respectively), neither significantly differs from zero (p_x = 0.262 and 0.134 respectively), and both overlap the global mean for birds ($p_{x[diff]}$ = 0.152 and 0.086 respectively).

Sexual selection has been reported to influence brain size within Charadriiformes – with differential effects among species belonging to polyandrous and monogamous social mating systems[1]. Shorebirds have high diversity in mating systems – notably showing almost all described systems and even multiple amongst populations of the same species[2]. Within Charadrii, this is likely to be linked with their unusually high diversity in sexual size dimorphism[3]. Whether this is a potential cause for the patterns we observe remains to be explored.

Summary

We provide a comparison between the estimated curvature for each of the groups discussed above to the global animal curvature in **Fig. S6.** These comparisons are for visual purposes only: global curves are estimated from an animal-wide model whereas sub-clade curves are from class-level analyses. However, a quick glance at this figure indicates that – potentially apart from Labriformes and Atlantogenata – that many of these subclades may still diverge from global curvature despite not differing from their class-specific curve.

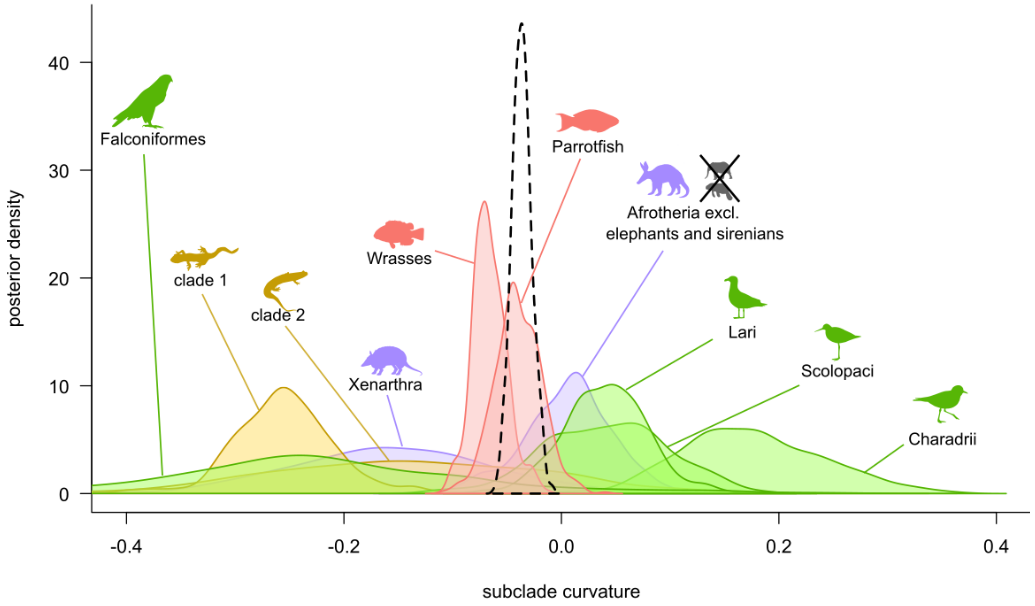

Fig. S6 Comparison of sub-clade curvature to global curvature after removing outliers and splitting into sub- sub-clades*. The posterior distribution of the estimated global quadratic parameter – dashed black line –observed across all species (N = 4676). The posterior distribution of quadratic parameters for each of the groups examined in detail are shown, coloured according to the class to which they belong.*

Supplementary Note 2: Significance of curvature

In our main text, we tested whether any group (class, sub-clade) significantly deviated from a global (grand mean) curvature estimated across all species. Here, we assess the significance of the parameters themselves. In our class curve model, we find a significantly positive slope and a median negative quadratic parameter in all vertebrate clades (**Table S1)**. The quadratic term for insects, testudines, and cartilaginous fish is non-significant (**Table S1)**, but these groups have small sample sizes compared with the other classes (N = 52, N = 25, and N = 57 respectively), and their curvature does not deviate from the global grand mean (**Fig. 1, inset**).

We determined whether the curvature of each class deviated from each other by calculating the difference between the estimated quadratic parameters at each iteration, creating a posterior distribution of differences in quadratic value between each group. We then assessed how often this distribution crossed zero ($p_{x[diff]}$). Where a difference overlaps zero in less than five percent of the posterior distribution (i.e. $p_{x[diff]} < 0.05$), two parameters are considered statistically distinct. In these comparisons (**Table S2**), we find that none of these three smaller groups (insects, cartilaginous fish, or testudines) differ from any other classes. That is, despite non-significant curves, they still do not significantly deviate from the curvature of any other group (**Table S2**).

Supplementary Note 3: Summary of rate heterogeneity

The variable rates models simultaneously estimate statistical parameters alongside heterogeneity in the rate of evolutionary change[4,5]. The variable rates regression method is a Bayesian Markov chain Monte Carlo model implemented within a PGLS framework that specifically identifies branch-level variation in the rate of evolution in the phylogenetically structured residual error of a regression model[4]. The method estimates a set of rate scalars *r* which define branch-wise shifts away from an underlying Brownian motion background rate of evolutionary change: where $r > 1$, a branch is evolving faster than the background rate of change, and where $0 \leq r < 1$, it is evolving slower. Regression parameters, background rate, and scalars are all estimated simultaneously. One way of summarizing these rates is to identify branches along which there has been historical *positive phenotypic selection (PPS)*, branches where the amount of expected change derived from shifts in the rate of evolution doubles that expected by the background rate of change ($r > 2$) – in at least 95% of the posterior distribution[4]. Similarly, *negative phenotypic selection (NPS*) can be defined along branches where the amount of expected change derived from shifts in the rate of evolution halves that expected by the background rate of evolution ($r < 0.5$) in at least 95% of the posterior distribution[4].

In our global curve analysis (N = 4679), there is substantial rate heterogeneity, with PPS observed in all animal groups (**Fig. S7**). A full list of all lineages, whether they are experiencing PPS or NPS, along with their median rate scalar are provided in **Supplementary Dataset S2.**

We identify PPS in ~7% of the lineages in the tree (N = 617). Most of the highest values of PPS occur along branches leading to individual species or small (N < 10) clades, notably including a very high rate along the lineage leading to our own species (*Homo sapiens*, with a rate ~ 44x the background). We also identify PPS in several rodent species – in line with recently published results from mammals[6]. Only 41 instances of PPS are identified along branches leading to larger clades of 10 or more species. These occur within the mammalian orders Rodentia (N = 8) and Carnivora (N = 1), the amphibian suborder Anura (n = 17), the fish clades Syngnathiformes (N = 1) and Ovalentaria (N = 111), along with passerine birds (N = 3), and the shorebird order, Charadriformes (N = 9). We identify no significant PPS in any other group comprising ten or more species.

NPS is much rarer, occurring along only 9 total branches in the whole phylogeny – all of which fall within the shorebird clade Charadriformes. Across all animals, the lowest rates are observed within non-avian reptiles – particularly within the squamate infraorder Iguania (the group including iguanas and chameleons). Many of the lineages within this group are evolving, on average, at ~25% of the rate observed across all other animals – although with variable posterior support. Other lineages with comparably low rates and stronger support (i.e., fulfilling both NPS criteria) include shorebirds (median rate scalar ~ 0.33), and bats – in particular, the horseshoe bats (genus: *Rhinolophus*) with median rate scalars of ~0.51. We provide a full list of all branches and their estimated rate of evolution in **Supplementary Dataset S2.**

The broad phylogenetic context within which the global curve analyses are placed means that phenotypic selection and all rate scalars are calculated and interpreted with respect to the background rate of evolutionary change observed across all animals. Therefore, a few large shifts deep in the tree can give rise to many subsequent downstream lineages in the tree evolving at rates different to the background[7] (e.g. the branch leading to mammals has a median scalar of 7.44, **Fig. S7**). To enable comparison within each animal group, we therefore also report the 10 highest and lowest rate scalars observed in each of the animal classes in **Tables S3-S9** (that are supported in >95% of the posterior distribution). These rates are not restricted to the magnitude criterion for positive phenotypic selection and so represent the fastest (and slowest) evolving species within each clade. All branches within testudines are evolving at the same rate (median r = 1) and so we do not include a separate table for this clade. Note that all this information can be derived from **Supplementary Dataset S2**.

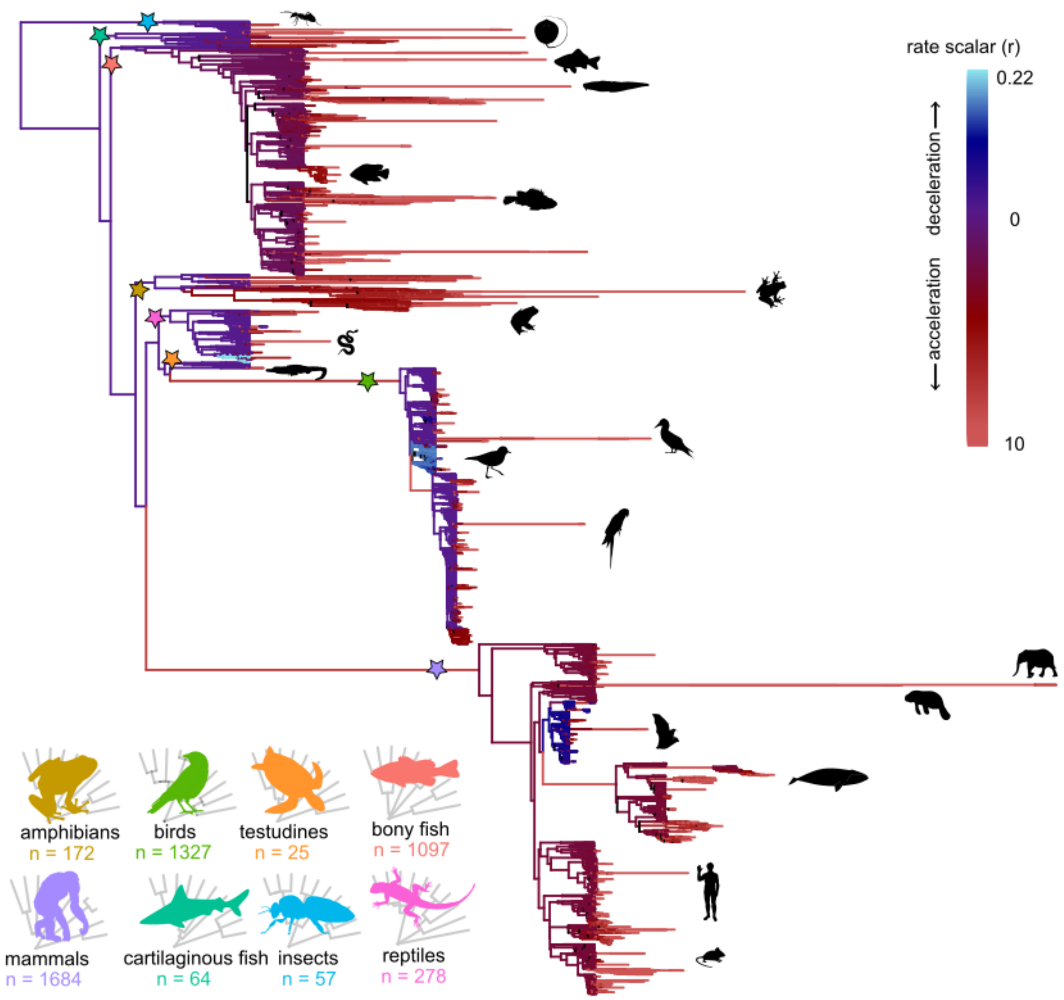

Fig. S7. Phylogenetic tree with branches scaled and coloured by rate of phenotypic evolution in the global curve model (N = 4679). *Coloured stars indicate the root of major clades. Silhouettes highlight various rate shifts. Median scalar (r) for each shift from top to bottom: Pogonomyrmex (harvester ants, r = 12.38), Himantura (whipray, r = 10.03), Carassius carassius (goldfish, r = 43.79), Acanthonus (bony-eared assfish, r = 25.71), Oreochromis (tilapia, r = 3.24), Sebastes (rockfish, median r = 19.83), Phyllomedusa bicolor (giant leaf frog, r = 55.67), Rana (true frogs, r = 4.15), Agkistrodon piscivorus (cottonmouth snake, r = 14.58), Crocodylus (true crocodiles, r=7.5), Morus (gannets, r = 31.21), Charadrius (typical plovers, r = 0.31), Cyanoramphus (parakeets, r = 30.63), Proboscidea (elephants, r = 17.02), Sirenia (manatees and dugongs, r = 17.11), Noctilio (bulldog bats, r = 11.00), Cetacea (whales and dolphins, r =13.72), Homo sapiens (humans, r = 44.05), Lemmus (true lemmings, r = 18.59). Silhouettes are for reference only; not to scale.* All *silhouettes are available from the public domain via phylopic.com.*

Supplementary Note 4: Bias in slope parameters without non-significant clades

Finally, we also tested the relationship between slope parameter and average body size from our subclade slope model (**Fig. 2C)** without cartilaginous fish, testudines, and insects to ensure they were not affecting the overall relationship. Without these clades, we still see a strong negative association between slope parameter and body size (r = -0.561, p <0.001, **Fig. S8**).

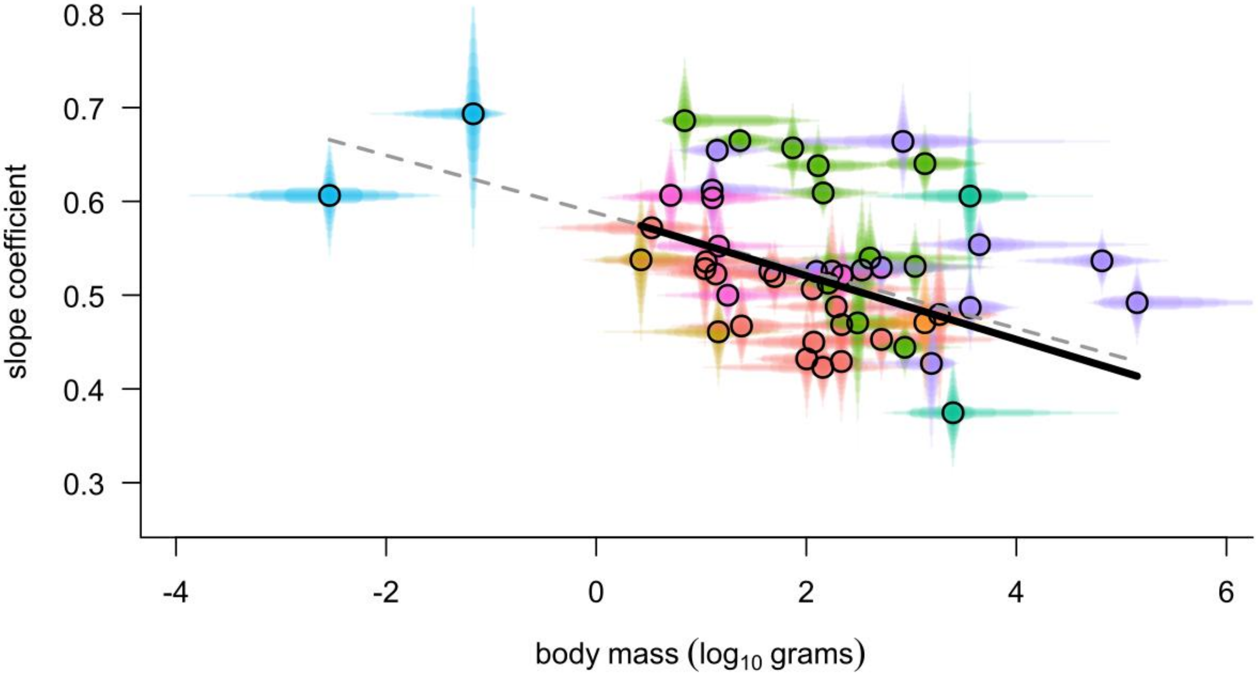

***Fig. S8 A negative relationship between linear slope parameters and body size across animal sub-clades (N = 51 subclades)**. Percentiles of the posterior distribution of slope parameters and body mass ranges are shown as transparent lines for each group. The dashed line shows the relationship including all groups; the solid black line shows the relationship excluding insects, sharks, and testudines.*

Supplementary Note 5: Results using a single-rate model of evolution

As described in our methods, we find substantial support for variable rates in all our PGLS analyses (using Bayes Factors). However, as it was not possible to estimate rate heterogeneity in all downstream analyses (e.g. mixed models, see below) we replicated our main PGLS analyses using models that assumed a constant rate of evolution (i.e. no rate heterogeneity).

We first repeated our global curve analysis. The single-rate global curve analysis retrieves a significantly negative quadratic parameter (median = -0.01, p_x = 0.000) and a positive slope (median = 0.55, p_x = 0.000). Therefore, across all animal groups and even in the absence of rate variation there is an overall curvature in the BBM in line with that reported for our analyses incorporating rate heterogeneity.

We then repeated our subclade curve analysis to test whether any one of 51 monophyletic subclades with N>20 taxa showed any departure from a global curvature estimated across all species. These are the same subclades as described for our main analysis. As in our main analysis, there is very little departure from the global curvature. We identify only 9 subclades as having a significantly different quadratic parameter to a global quadratic parameter estimated across all animal species (**Table S9**).

As we did for our main results, we investigated why we may have observed deviations from a global curvature by separating each subclade into two or more monophyletic groups (sub-subclades) based on phylogenetic relationships and then running a modified version of our subclade curve model using the procedure outlined above for our variable rates models. We also tested the effect of outlier taxa. The sub-subclades for all taxa are as defined in **Supplementary Dataset S1.**

Note that this includes three of the four subclades identified in our variable rates analysis (**Fig. S9:** Charadriformes, Atlantogenata, and Labriformes – here, we find no evidence for a significantly different curvature within Urodela). These and all other identified subclades are explored as described for our variable rates model. For the clades overlapping with our variable rates analyses, we do not re-plot the data and phylogenetic trees and simply plot the distributions of parameters; we therefore combine these into a single multi-panelled figure (**Fig. S9**).

Atlantogenata

In our single-rate mammalian sub-subclade curve model, Xenarthra exhibits a negative curve (median quadratic coefficient = -0.155) that does not differ from a global mammalian curvature ($p_{x[diff]}$ = 0.152). In contrast, Afrotheria is positive (median quadratic coefficient = 0.057) and remains distinct from other mammals($p_{x[diff]}$ = 0.000, **Fig. S9a**). However, when the model is repeated excluding Sirenia and Proboscidea, this effect disappears ($p_{x[diff]}$ = 0.156, **Fig. S9**).

Charadriiformes

Within shorebirds, we find that none of the three sub- sub-clades show curvature that deviates from the global mammal curve – although they do all tend towards positive parameter values. However, in this analysis, we find that all three groups have very wide estimates for the observed curvature, implying large rate heterogeneity in this group that may be linked to their differences (**Fig. S9b**).

Falconiformes

When the two small-bodied species are removed from the analysis, the curvature for Falconiformes is highly similar (median quadratic coefficient = -0.232) and remains statistically distinct from the global bird curve ($p_{x[diff]}$ = 0.002, **Fig. S9c**).

Labriformes

When we study parrotfish and wrasses separately, both clades have generally negative curvature and do not differ from a global curvature estimated across all fish (median quadratic coefficient = -0.0317, p_x = 0.454 and median quadratic coefficient = -0.083, p_x = 0.072 for parrotfish and wrasses respectively, see **Fig. S9d**). The result is indistinguishable after the exclusion of the potential outliers.

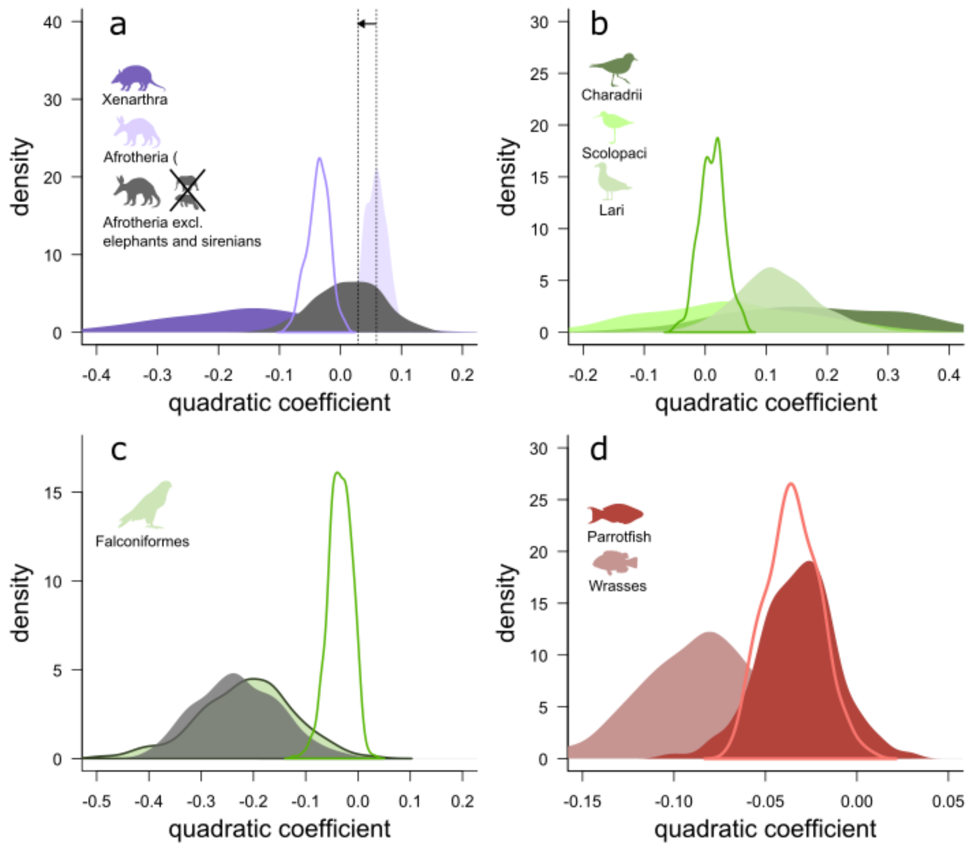

Fig. S9 Exploring the curvature within Atlantogenata, Charadriformes, Falconiformes, and Labriformes for single-rate models. *Sample sizes are as in the corresponding variable rates models. In each panel, the difference in estimated quadratic parameter is shown compared to the grand mean curvature estimated across all other members of the larger class using a single-rate model. The class-level curvature is indicated by a hollow bold-outlined distribution. In (a), we compare the curvature in Xenarthra and Afrotheria to all mammals. The dark grey distribution shows the estimated relationship excluding the large-bodied sirenians and elephants. Note that removing these few species shifts the distribution (median values indicated by the dotted vertical lines) such that the distribution no longer diverges from the grand mean curvature. In (b) we compare the three-subclades of Charadriformes to the grand mean curvature estimated across all birds. All three distributions are widely estimated and substantially overlap the global mean. In (c), we show the difference in the Falconiformes quadratic parameter both before (green) and after (grey) excluding potential outliers. In (d), we compare the curvature across all fish to that within parrotfish and wrasses; neither of which significantly differ.*

Psittaciformes

Psittaciformes is an order of brightly coloured birds including parrots and parakeets – characterized by remarkable intelligence. In this clade, we find significantly positive curvature (median quadratic coefficient = 0.101, p_x = 0.033) that substantially deviates from the global avian curve ($p_{x[diff]}$ = 0.005). However, when we split Psittaciformes into the three major families (cockatoos of the family Cacatuidae, N = 13, new-world parrots or Psittacidae, N = 27, and old-world parrots or Psittaculidae, N = 46), we find that the positive curvature is entirely driven by an extraordinarily high curvature in new-world parrots (**Fig. S10**).

Both new-world and old-world parrots significantly differ from the global bird curve ($p_{x[diff]}$ = 0.038 and 0.000 respectively). However, they differ in opposite directions, with new world parrots having a median quadratic parameter = 0.340 and old-world parrots with a median quadratic parameter = -0.315. Cockatoos, on the other hand, whilst having on average a negative curvature (median quadratic coefficient = -0.185), do not differ from the curvature observed in other birds ($p_{x[diff]}$ = 0.06). These results are visualized in **Fig. S10.**

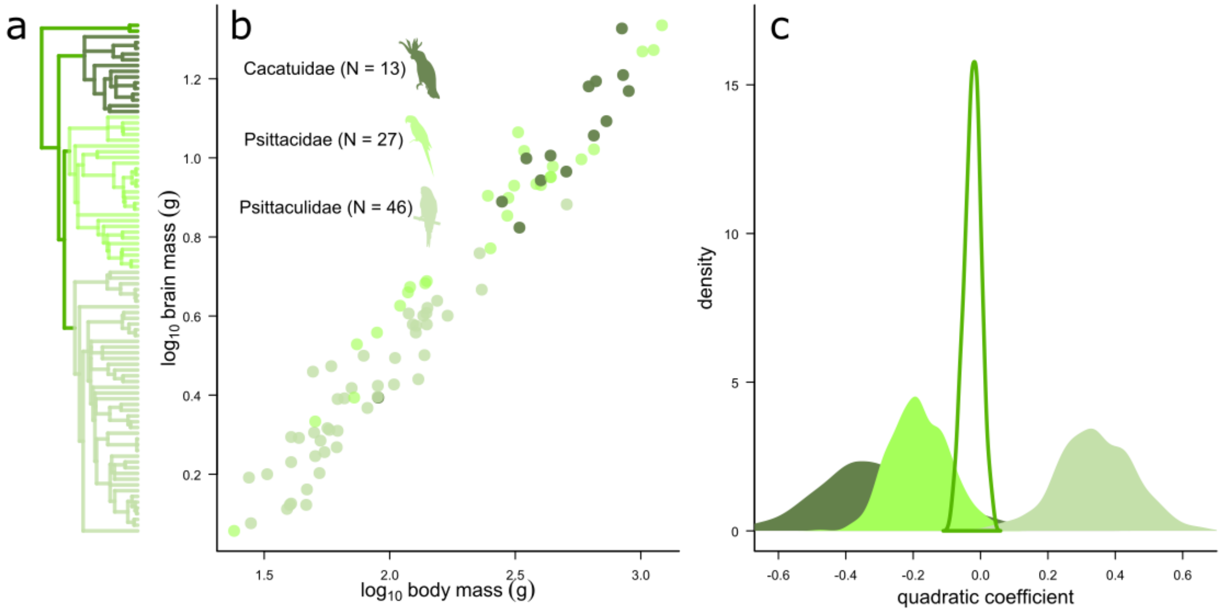

Fig. S10 Exploring the curvature within Psittaciformes. *(a) The phylogenetic structure of the group is shown, with branches coloured by assignment to four major monophyletic sub-clades. (b) Brain and body size data for Psittaciformes, coloured by sub-clade (excluding the New Zealand Parrots with N = 2). (c) Difference in estimated quadratic parameter for each of the three sub-clades large enough for further study compared to the grand mean curvature estimated across all birds (solid green outline distribution).*

Ovalentaria

Ovalentaria is a large clade of ray-finned fishes that is variable in definition. We used NCBI[8] to define taxonomic groups within fish as this source aligned with the structure of the phylogenetic tree. Ovalentaria was defined using a combination of monophyletic groups that formed a single clade – facilitating inclusion of many species groups that would otherwise have been excluded owing to small sample sizes. However, in our dataset, there are several clades that fall within the definition of Ovalentaria that are also large enough to be explored independently. These are the Blenniformes (N = 27) which includes blennies and related groups; the Cichliformes (N = 83) which is often considered a separate order of fish comprising cichlids, blennies and leaffish; the Beloniformes (N = 14) which includes flying fish and other surface-feeding fish; and the large family Pomacentridae (N = 63) which includes damselfish and clownfish.

Among Ovalentaria, only Blenniiformes (the order of fish referred to as blennies) is identified as deviating from a global curvature (median difference = 0.16, $p_x = 0.04$, **Fig. S11**). No other sub-subclade shows any deviation (all $p_{x[diff]} > 0.05$). Thus, the apparent positive curvature identified in Ovalentaria is only driven by blennies, which has a median quadratic parameter of 0.158.

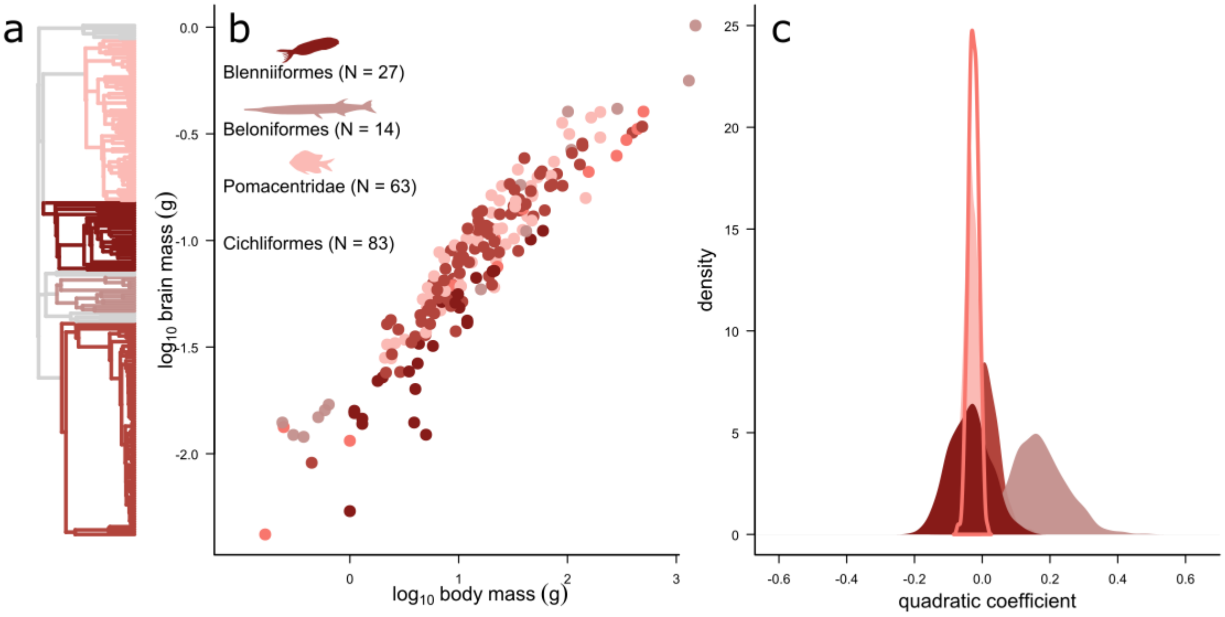

Fig. S11 Exploring the curvature within Ovalentaria. *(a) Phylogeny of Ovalentaria, with branches coloured by assignment to four main sub-clades – all other remaining lineages are shaded grey. (b) Brain and body size data for the four major sub-clades. (c) Difference in estimated quadratic parameter for each of the four sub-clades compared to the grand mean curvature estimated across all fish (solid coral outline distribution).*

Carangiformes

Carangiformes, an order of ray-finned fishes including jack mackerels, flounders and scads, follows the same pattern as the global curvature (a negative quadratic, **Table S9**), but with a steeper curve. There are no clear subdivisions in this subclade and no obvious outliers – and so it remains unclear what the cause of this difference could be (**Fig. S12**).

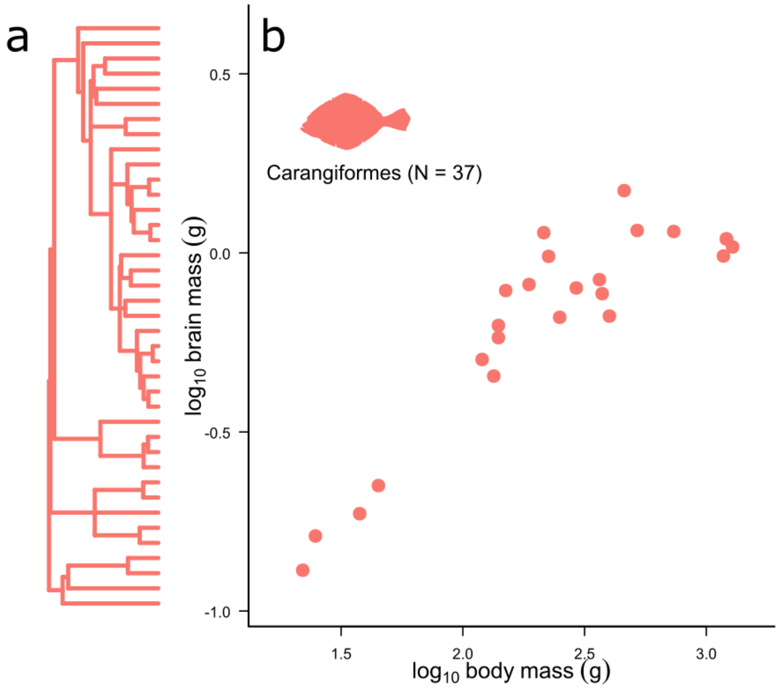

Fig. S12 Variation in brain and body size among Carangiformes. *(a) The phylogenetic structure of the group. (b) Brain and body size data for Carangiformes.*

Syngnathiformes

Syngnathiformes is an order of ray-finned fish that includes seahorses, pipefish and allies. In our dataset, we used NCBI[8] to assign species to taxonomic groups as these groups tended to align with the structure of the phylogeny. However, this resulted in a subclade of this group (goatfish and dragonets) being assigned to Syngnathiformes despite being considered a separate order (Mulliformes) in other sources (e.g. Fishbase[9]). Mulliformes, along with another large clade of Syngnathiformes fall in a basal polytomy along with a small group of species (N =3) known as trumpet fish and cornet fish (**Fig. S13**). These species are unusually and extremely elongated but are considered members of Syngnathiformes. There is one very tiny pipefish which seems to have a much larger brain size than would be expected from its body size (*Duckerocampus dactyliophorus*) which is a potential outlier (**Fig. S13**).

We find that – without the outlier – the clade still significantly differs from the global fish mean ($p_{x[diff]}$ =0.032, **Fig. S13**), with an overall marginally positive curve (median quadratic coefficient = 0.016) although this does not statistically differ from zero (p_x = 0.188). When we divide the clade into Syngnathiformes and Mulliformes, it becomes apparent that Mulliformes does not diverge from the global fish curvature (p_x = 0.06) and rather the positive curvature is driven by the seahorses and pipefish ('true' Syngnathiformes). In this clade, the curvature is significantly positive (median quadratic coefficient = 0.13, p_x = 0.024) and significantly differs from the curvature estimated across the rest of fish ($p_{x[diff]}$ = 0.002). Whilst it yet remains unclear why this might happen within seahorses and pipefish, it should be noted that several of the ~300 rate shifts observed in clades of species where N>10 taxa occur within this group in our variable rates analyses.

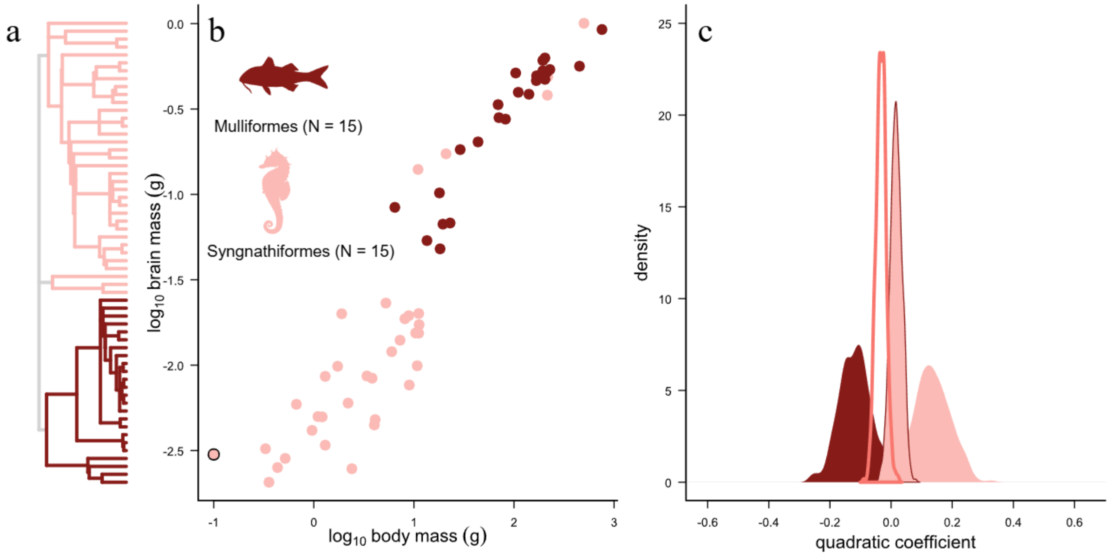

Fig. S13 Exploring the curvature within Syngnathiformes. *(a) The phylogenetic structure of the group is shown, with branches coloured by assignment to two major sub-clades – all other remaining lineages are shaded grey. (b) Brain and body size data for the major clades – the potential outlier is outlined in black. (c) Difference in estimated quadratic parameter for each of the sub-clades large enough for further study compared to the grand mean curvature estimated across all fish (solid coral outline distribution). The quadratic parameter estimated across all Syngnathiformes (excluding the outlier) is shown as an outlined pale distribution. Image credit to Emilie Boulanger for the Mullus surmuletus silhouette via the CC-BY 3.0 license (https://creativecommons.org/licenses/by/3.0/).*

Perciformes

Perciformes are a large order (or superorder in some sources) of fish that are described as "perch-like", including species like sea bass and perch. Amongst these species there are three potential outliers – two very small, and one very large (**Fig. S14**). There are also two large monophyletic subclades that could be tested in isolation: the first comprises a number of different groups including groupers, sea basses and anthias (N = 39), and the second comprises a number of groups that are now more commonly considered to be members of the modern order Scorpaeniformes (N = 51)[9].

When we study the relationships amongst Perciformes **–** only Scorpaeniformes of the two clades shows any significant deviation from the global curvature ($p_{x[diff]}$ = 0.000, median quadratic coefficient = 0.039, **Fig. S14**). This curvature is significantly positive ($p_{x[}$ = 0.016). What exactly is happening in this subclade is worth further exploration in combination with observed rate heterogeneity.

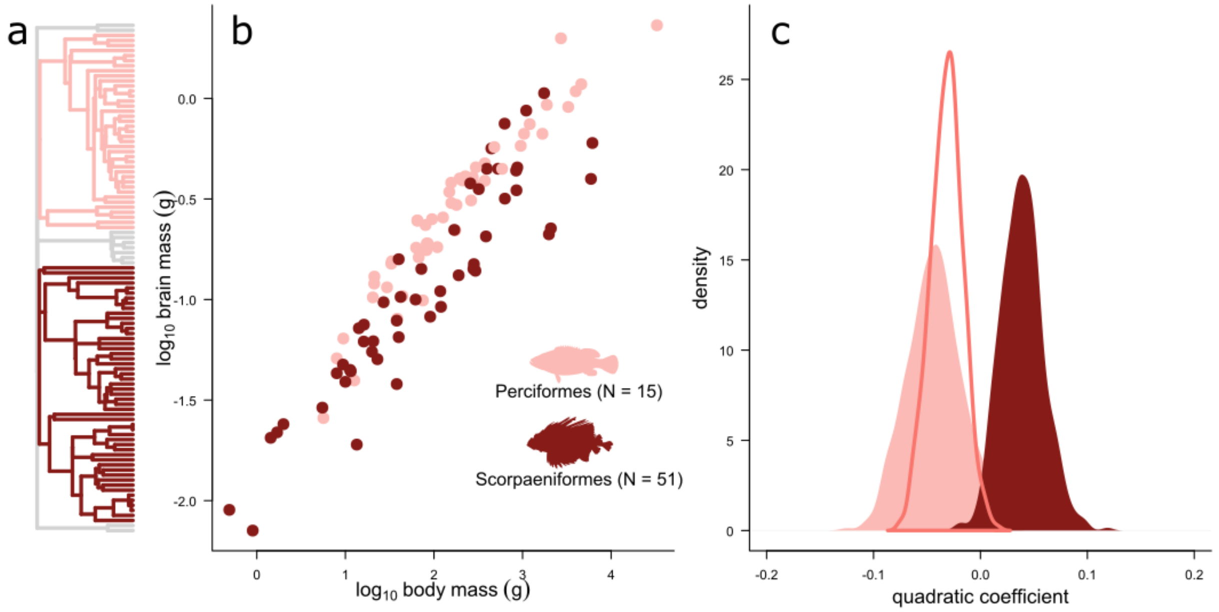

Fig. S14 Exploring the curvature within Perciformes. *(a) The phylogenetic structure of the group is shown, with branches coloured by assignment to two major sub-clades – all other remaining lineages are shaded grey. (b) Brain and body size data for the major clades. (c) Difference in estimated quadratic parameter for each of the two sub-clades large enough for further study compared to the grand mean curvature estimated across all fish (solid coral outline distribution).*

Subclade slope analysis

Finally, we also ran a single-rate version of our subclade slope analysis (in which we estimate a separate intercept and slope for each of the 51 subclades). In this model and in line with the results reported in the main text, we find a significant negative association between the slope of the BBM relationship from a *subclade slope model* estimating a separate intercept and slope for each subclade and the average body size across all fifty-one subclades (α = 0.559, p <0.001; β = -0.023, p = 0.021). This relationship persists to the exclusion of the small-bodied insects (α = 0.568, p < 0.001; β = -0.028, p = 0.013), and to the exclusion of all non-significant groups (insects, sharks, and testudines, α = 0.567, p < 0.001; β = -0.031, p = 0.009).

Supplementary Note 6: Theoretical expectations derived from within-species allometry

Within individual species, the relationship between brain and body size can be expressed as a simple linear equation:

$$brain_{i,j} = \alpha + \beta_i body_{i,j} + \epsilon_{i,j} \qquad \textbf{\textit{Eq.S1}}$$

A size-dependency observed in individual brain allometries (i.e., a negative association between average body size $\overline{body_i}$ and species-level slopes β_i, Figure 4a inset) can be expressed as a linear effect and incorporated into **Eq. S1** as follows:

$$brain_{i,j} = \alpha + (\gamma_0 + \gamma_1 \overline{body_i}) body_{i,j} + \epsilon_{i,j} \qquad \textbf{\textit{Eq.S2}}$$

, where the within-species slope (β_i) is derived from the slope γ_1 and intercept γ_0 of the relationship between β_i and $\overline{body_i}$. When we substitute **Eq. S2** into the context of an across-species relationship between species-level averages, we see quadratic curvature (Figure 4):

$$\overline{brain_i} = \alpha + \gamma_0 \overline{body_i} + \gamma_1 \overline{body_i}^2 + \epsilon_{i,j} \qquad \textbf{\textit{Eq.S3}}$$

The key insight here is that the across-species relationship includes both a linear term and a second-order quadratic term owing to the negative association between γ_1 and $\overline{body}$. That is, the strength of an overall across-individual relationship decreases as body size increases across individuals. Although quadratic terms imply a downward curve at larger sizes, such a downturn does not occur in the observed range of animal body sizes (Figure 2) and it has recently been demonstrated that second order polynomials provide indistinguishable results from power curves in the context of brain size evolution[6].

Supplementary Note 7: Body size dependency in within-species allometry

In our main text, we extracted brain and body size data adjusted for sex and age for a total of 376 vertebrate species with at least 10 individual measurements from Tsuboi et al.[10]. Consistent with our hypothesis, using phylogenetic generalized least squares models[11], we identified a strongly significant negative relationship between the within-species slopes of the BBM relationship and the average body size of each species (**Fig. 4A**, $\alpha = 0.37$ [$p < 0.001$]; $\beta = -0.04$ [$p = 0.008$]; $\lambda = 0.13$). We find no such association with average body size and the significance of the within-species slope ($\alpha = 0.181$ [$p = 0.005$]; $\beta = -0.013$ [$p = 0.457$]; $\lambda = 0.151$), the mean-squared error of the slope parameter ($\alpha = 0.37$ [$p < 0.001$]; $\beta = -0.04$ [$p = 0.008$]; $\lambda = 0.13$), or the R^2 of the slope ($\alpha = 0.447$ [$p < 0.001$]; $\beta = 0.028$ [$p = 0.106$]; $\lambda = 0.564$).

Supplementary Note 8: The effect of common brain size covariates on size-dependency

If some aspect of brain size evolution is being limited at larger sizes, it is logical to turn to common correlates of brain size to determine whether they have some impact on the curvature. That is, if the mass dependency we observe reflects how particular functions, behaviours, or ecologies are conserved across a range of animal sizes, we would expect to no longer see curvature in a model that incorporates such factors.

Previous tests in mammals revealed that neural connectivity, axon size scaling and myelination had no effect on the curvilinearity observed in the mammalian brain and body size relationship[6]. Similarly, despite the possibility that large species may be unable to sustain the high cost of their large brains, the incorporation of metabolic rates into the mammal-wide analysis had no effect[6], although there is a significant effect on brain size beyond body size[6,14].

Here, we tested the possibility that the brain size curvature was being driven by any of three alternative brain size correlates commonly reported to be associated with brain size at the species level including, neural connectivity and density[15,16], diet[17], and environmental temperature[18]. For each trait, we collected data from published literature. We then repeated our global curve analysis in the subset of species limited to the trait of interest. Finally, we tested the impact of including the trait of interest as an additional covariate in our global curve model.

For environmental temperature, we used data from a recently published analysis of Mesozoic dinosaurs and mammaliaforms[19]. From this dataset, we obtained the average temperatures for a species natural range measured in Kelvin for a total of 2,620 mammals and birds that overlapped with our brain size data. In a global curve model restricted to these taxa, we still retrieve a significant negative curvature (median quadratic parameter = -0.024, $p_x = 0.00$) with a positive slope (median = 0.71, $p_x = 0.00$). When we include $\log_{10}$ temperature as an additional

covariate, we see no change in the value or significance of these parameters – although we do note with interest that the effect of temperature itself is significantly negative (median $\beta = -0.24$, $p_x = 0.04$).

For diet, we used data from EltonTraits 1.0[20]. From this dataset, we obtained the dietary composition for a total of 2,938 mammal and bird species that overlapped with our brain size data. We assigned species according to the dominant food source: (i) invertebrates, (ii) fruit and nectar, (iii) seed and leaf, (iv) vertebrates including fish and scavengers, and (v) where no food source comprised >50% of the diet, a species was considered omnivorous. In a global curve model restricted to these taxa, we still retrieve a significant negative curvature (median quadratic parameter = -0.014, $p_x = 0.00$) with a positive slope (median = 0.67, $p_x = 0.00$). When we allow each of the dietary categories to have an independent slope in the relationship between brain and body size (i.e. we include both an intercept and cross-product for each category as an additional covariate), the results are qualitatively identical. We find significant negative curvature (median quadratic parameter = -0.02, $p_x = 0.00$) with a positive slope (median = 0.70, $p_x = 0.00$) even in the face of significant differences in the relationship between brain and body size among species with different diets (**Table S12**).

Supplementary Note 9: Analyses incorporating uncorrected within-species variation

In our main analyses, we centred data according to sex (male, female, or unreported) and measurement method (volume or mass) as in the original paper [10]. However, our major conclusions are not affected if we do not perform this correction.

Using phylogenetic generalized linear mixed models[12] (PGLMMs) to account for individual-level variation in both brain and body size, we recover a significant negative quadratic parameter across species (median = -0.035, $p_x = 0.000$) as well as a significant positive slope (median = 0.54, $p_x = 0.000$). Using the hierarchical model (a PGLMM model using within-group centring[13], we no longer find any curvature across species (mean $\beta = 0.005$, $p_x = 0.3218$).

As in the corrected data, the relationship acting within individual species is generally much shallower (mean $\beta = 0.42$, $p_x = 0.000$) than that observed across species (mean $\beta = 0.59$, $p_x = 0.000$).

Table S1. Parameters from the class curve model, converted to the estimated intercept, slope, and quadratic values for each class. The significance of each (as the proportion of the posterior distribution crossing zero, p_x is shown in square brackets).

Subclade	Intercept	Slope	Quadratic
**Bony fish**	-1.88 [p_x = 0.00]	0.61 [p_x = 0.00]	-0.03 [p_x = 0.00]
**Amphibians**	-2.06 [p_x = 0.00]	0.57 [p_x = 0.00]	-0.05 [p_x = 0.02]
**Birds**	-1.33 [p_x = 0.01]	0.78 [p_x = 0.01]	-0.04 [p_x = 0.00]
**Cartilaginous fish**	-1.16 [p_x = 0.05]	0.51 [p_x = 0.05]	-0.01 [p_x = 0.13]
**Insects**	-2.35 [p_x = 0.00]	0.55 [p_x = 0.00]	-0.02 [p_x = 0.3]
**Mammals**	-1.00 [p_x = 0.03]	0.67 [p_x = 0.00]	-0.02 [p_x = 0.00]
**Squamates**	-1.82 [p_x = 0.00]	0.66 [p_x = 0.00]	-0.04 [p_x = 0.00]
**Testudines**	-2.18 [p_x = 0.01]	0.82 [p_x = 0.03]	-0.05 [p_x = 0.18]

*Table S2. Pairwise comparison of class-level quadratic parameters from our class curve model (act = bony fish, amp = amphibians, ave = birds, cho = bony fish, ins = insects, mam = mammals, squ = squamates, and tes = testudines). In the top right half of the table, the median difference between the estimated quadratic parameters of each group is reported (row-column). In the bottom left half of the table, the proportion of the posterior distribution of differences between the two estimated quadratic parameters (column-row) is reported. *Where this value <0.05, two parameters are considered to be different from one another.*

β / p_x	**act**	**amp**	**ave**	**cho**	**ins**	**mam**	**squ**	**tes**
**act**		0.02	0.01	-0.02	-0.01	-0.01	0.00	0.02
**amp**	0.22		-0.21	0.07	0.03	-0.10	-0.09	-0.25
**ave**	0.17	0.00*		0.27	0.23	0.11	0.12	-0.05
**cho**	0.31	0.42	0.20		-0.04	-0.16	-0.15	-0.31
**ins**	0.29	0.42	0.06	0.45		-0.12	-0.11	-0.27
**mam**	0.01*	0.05	0.00*	0.30	0.18		0.01	-0.15
**squ**	0.34	0.10	0.00*	0.32	0.20	0.38		-0.15
**tes**	0.39	0.27	0.46	0.25	0.25	0.36	0.34	

Table S3. The largest and smallest rate scalars observed within mammals. The number of descendants arising from each branch is listed; where this is greater than 2, a brief description is given in the taxa column, otherwise the species names are given. We report the median scalar along with the proportion of the posterior distribution in which each branch is scaled.

**Taxa**	**N**	**subclade(s)**	**scalar**	**%**
Mustela putorius	1	Carnivora	37.855	10
Meriones shawi	1	Rodentia	38.754	10
Zapus princeps, Zapus trinotatus	2	Rodentia	39.5	10
Homo sapiens	1	Primates	44.052	10
Ferungulata	38	Cetartiodactyla and	45.13	10
Meriones unguiculatus	1	Rodentia	46.136	10
Ateles marginatus	1	Primates	53.043	10
Arctocephalus galapagoensis	1	Carnivora	74.478	10
Lemmus lemmus	1	Rodentia	114.13	10
Lemmus sibiricus	1	Rodentia	125.51	10
Rhinolophus paradoxolophus	1	Chiroptera	0.45	10
Rhinolophus malayanus	1	Chiroptera	0.458	10
Rhinolophus acuminatus	1	Chiroptera	0.458	10
Rhinolophus macrotis	1	Chiroptera	0.458	10
Rhinolophus megaphyllus	1	Chiroptera	0.461	10
Rhinolophus macrotis, Rhinolophus	2	Chiroptera	0.464	10
Rhinolophus species (N = 5)	5	Chiroptera	0.466	10
Rhinolophus species (N = 4)	4	Chiroptera	0.474	10
Cormura brevirostris	1	Chiroptera	0.494	10
Rhinolophus lepidus	1	Chiroptera	0.5	10

Table S4. The largest and smallest rate scalars observed within bony fish. The number of descendants arising from each branch is listed; where this is greater than 2, a brief description is given in the taxa column, otherwise the species names are given. We report the median scalar along with the proportion of the posterior distribution in which each branch is scaled. Note that the lowest rates in this clade have a scalar = 1; these branches are evolving at the background rate across all animals. There are 12 branches evolving at this rate; all are included here.

**Taxa**	**N**	**subclade(s)**	**scalar**	**%**
Alosa immaculata	1	Otocephala	28.946	100
Gymnothorax pictus	1	Elopomorpha	29.719	100
Alosa caspia	1	Otocephala	30.152	100
Meiacanthus nigrolineatus	1	Ovalentaria	30.399	99.8
Scorpaenodes guamensis	1	Perciformes	30.444	99.8
Alosa caspia, Alosa immaculata	2	Otocephala	31.086	100
Carassius carassius	1	Otocephala	43.79	100
Arothron nigropunctatus	1	Tetraodontiformes	47.185	100
Tanganicodus irsacae	1	Ovalentaria	57.159	100
Corythoichthys intestinalis	1	Syngnathiformes	62.009	100
All bony fish	1097	All bony fish subclades	1	17.4
All bony fish except sturgeons	1095	All bony fish subclades	1	25.6
All bony fish except sturgeons and gars	1093	All bony fish subclades	1	45.8
Amia calva, Lepisosteus osseus	2	NA (gars and bowfins)	1	49.4
Lepisosteus osseus	1	NA (gar)	1	47.2
Amia calva	1	NA (bowfin)	1	47
Acipenser transmontanus,	2	NA (sturgeons)	1	27.2
Acipenser transmontanus	1	NA (sturgeons)	1	26
Scaphirhynchus platorynchus	1	NA (sturgeons)	1	25
Polypterus palmas buettikoferi, Polypterus	2	NA (bichirs)	1	17.8
Polypterus palmas polli	1	NA (bichirs)	1	18.4
Polypterus palmas buettikoferi	1	NA (bichirs)	1	18.6

Table S5. The 10 largest rate scalars observed within amphibians. The number of descendants arising from each branch is listed; where this is greater than 2, a brief description is given in the taxa column, otherwise the species names are given. We report the median scalar along with the proportion of the posterior distribution in which each branch is scaled. Note that the lowest rates in this clade have a scalar = 1; these branches are evolving at the background rate across all animals. There are 64 branches evolving with this rate amongst amphibians; we therefore do not include these branches in this table for brevity.

**Taxa**	**N**	**subclade(s)**	**scalar**	**%**
Pelophylax lessonae	1	Anura	19.285	100
Amolops lifanensis	1	Anura	20.008	100
Polypedates megacephalus	1	Anura	22.051	100
Oophaga pumilio, Oophaga speciosa	2	Anura	24.121	100
Oophaga speciosa	1	Anura	26.151	100
Amolops granulosus	1	Anura	31.218	100
Phyllomedusa bicolor	1	Anura	55.667	100
Atelopus chiriquiensis	1	Anura	75.733	100
Polypedates leucomystax	1	Anura	76.731	100
Oophaga pumilio	1	Anura	83.324	100

Table S6. The largest rate scalars observed within cartilaginous fish. The number of descendants arising from each branch is listed along with the species names. We report the median scalar along with the proportion of the posterior distribution in which each branch is scaled. Note that the lowest rates in this clade have a scalar = 1; these branches are evolving at the background rate across all animals. There are 86 branches evolving with this rate amongst cartilaginous fish; we therefore do not show these here.

**Taxa**	**N**	**subclade(s)**	**scalar**	**%**
Scymnodon ringens	1	sharks	4.441	95
Mustelus antarcticus, Mustelus	2	sharks	4.476	79.4
Somniosus microcephalus	1	sharks	4.719	96
Rajella fyllae	1	rays	5.085	74.6
Isistius brasiliensis	1	sharks	5.252	96.6
Mustelus lenticulatus	1	sharks	7.03	87.6
Himantura, Potamotrygon motoro	2	rays	7.926	80.8
Mustelus antarcticus	1	sharks	8.392	92
Himantura	1	rays	10.029	82.4
Potamotrygon motoro	1	rays	18.695	99.2
Scymnodon ringens	1	sharks	4.441	95

Table S7. The largest rate scalars observed within insects. The number of descendants arising from each branch is listed; where this is greater than 2, a brief description is given in the taxa column, otherwise the species names are given. We report the median scalar along with the proportion of the posterior distribution in which each branch is scaled. Note that the lowest rates in this clade have a scalar = 1; these branches are evolving at the background rate across all animals. There are 97 branches evolving with this rate amongst insects; we therefore do not show these here.

**Taxa**	**N**	**subclade(s)**	**scalar**	**%**
Apis mellifera	1	bees	7.937	89.4
Pogonomyrmex colei, Pogonomyrmex rugosus	2	ants	10.169	68.4
Pogonomyrmex rugosus	1	ants	12.381	70.2
Acromyrmex octospinosus, Acromyrmex versicolor, Atta	4	ants	15.119	98
Acromyrmex versicolor	1	ants	15.139	98.8
Acromyrmex octospinosus	1	ants	15.217	99.8
Acromyrmex octospinosus, Acromyrmex versicolor	2	ants	15.37	99
Trachymyrmex cornetzi	1	ants	15.447	97.8
Atta texana	1	ants	17.757	100
Pogonomyrmex colei	1	ants	22.191	96.8
Apis mellifera	1	bees	7.937	89.4

Table S8. The 10 largest and smallest rate scalars observed within non-avian reptiles. Note that we include crocodilians in this table owing to their small number; all testudines have a rate scalar of 1 and are not included. The number of descendants arising from each branch is listed; where this is greater than 2, a brief description is given in the taxa column, otherwise the species names are given. We report the median scalar along with the proportion of the posterior distribution in which each branch is scaled.

**Taxa**	**N**	**subclade(s)**	**scalar**	**%**
Carlia bicarinata	1	Scincomorpha	6.642	77.2
Lygisaurus foliorum, Lygisaurus	2	Scincomorpha	7.362	82
Crocodylus niloticus	1	NA (Nile crocodile)	7.373	68
Lygisaurus novaeguineae	1	Scincomorpha	7.53	83
Crocodylus acutus	1	NA (American crocodile)	7.565	67.4
Lialis jicari	1	Gekkonata	8.463	88.8
Lialis burtonis	1	Gekkonata	8.971	91.2
Lerista	4	Scincomorpha	9.826	90.6
Lygisaurus foliorum	1	Scincomorpha	10	99
Agkistrodon piscivorus	1	Serpentes	14.58	99.2
Bronchocela cristatella	1	Iguania	0.222	87.6
Branch within Iguania	11	Iguania	0.222	86.4
Ctenophorus gibba	1	Iguania	0.222	89.8
Acanthosaura armata	1	Iguania	0.223	88.4
Calotes versicolor	1	Iguania	0.223	88
Ctenophorus	14	Iguania	0.223	88.6
Ctenophorus cristatus	1	Iguania	0.224	90
Ctenophorus nuchalis	1	Iguania	0.224	88.2
Acanthosaura armata, Acanthosaura	3	Iguania	0.224	88.2
Ctenophorus	13	Iguania	0.224	89.2
Ctenophorus gibba	1	Iguania	0.224	89.8
Ctenophorus cristatus	1	Iguania	0.224	90
Ctenophorus rufescens	1	Iguania	0.224	90.2
Ctenophorus nuchalis	1	Iguania	0.224	88.2

Table S9. The 10 largest and smallest rate scalars observed within birds. The number of descendants arising from each branch is listed; where this is greater than 2, a brief description is given in the taxa column, otherwise the species names are given. We report the median scalar along with the proportion of the posterior distribution in which each branch is scaled.

**Taxa**	**N**	**subclade(s)**	**scalar**	**%**
Eudyptes chrysolophus	1	Aequo 1	18.865	99.8
Eudocimus albus	1	Aequo 1	19.03	78.2
Eudocimus ruber	1	Aequo 1	23.33	87
Morus bassanus, Morus serrator	2	Aequo 1	25.72	97.4
Cyanoramphus	3	Psitt	30.611	99.4
Cyanoramphus unicolor	1	Psitt	30.631	99.4
Morus bassanus	1	Aequo 1	36.693	97.8
Cyanoramphus auriceps	1	Psitt	37.166	100
Cyanoramphus novaezelandiae	1	Psitt	44.531	100
Morus serrator	1	Aequo 1	64.119	99.8
Species within *Charadrius*	5	Aequo 2	0.303	100
Charadrius pecuarius	1	Aequo 2	0.303	100
Charadrius alexandrinus	1	Aequo 2	0.303	100
Charadrius ruficapillus	1	Aequo 2	0.303	100
Species within *Charadrius*	6	Aequo 2	0.303	100
Species within *Charadrius*	9	Aequo 2	0.303	100
Charadrius bicinctus	1	Aequo 2	0.303	100
Species within *Charadrius*	7	Aequo 2	0.304	100
Charadrius leschenaultii	1	Aequo 2	0.304	100
Charadrius wilsonia	1	Aequo 2	0.305	100

Table S10. Clades identified as deviating from global curvature in a single-rate model. Clades marked with an asterisk are not significantly different from the grand mean curvature estimated within their class after removing outliers or amongst their substituent subclades (see Supplementary Text).

Subclade	N	Median Difference	p_x
**Ovalentaria**	199	0.034	0.032
**Syngnathiformes**	59	0.055	0.001
**Perciformes**	101	0.029	0.04
**Carangiformes**	39	-0.056	0.024
**Labriformes***	95	-0.027	0.024
**Charadriiformes***	140	0.143	0.007
**Falconiformes**	24	-0.196	0.009
**Psittaciformes**	89	0.127	0.005
**Atlantogenata***	47	0.08	0.000

Table S11. Pairwise comparison of intercepts (A) and slopes (B) of the relationship between brain and body size for species assigned to different dietary categories (based on the proportion of food types observed). In the top right half of the table, the median difference between the estimated parameters for each dietary category is reported (row-column). In the bottom left half of the table, the proportion of the posterior distribution of differences between the two estimated parameters (row-column) is reported. Where this value <0.05, two parameters are considered to be significantly different from one another. Note that these parameters are from a model that simultaneously estimates an overall curvature across all bird and mammal species (N = 2938).

(A)

**6** / **p_x**	**Fruit & Nectar**	**Invertebrates**	**Omnivores**	**Plants (Other)**	**Vertebrates. & Scavengers**
**Fruit & Nectar**		0.00	-0.05	-0.03	-0.02
**Invertebrates**	0.47		-0.05	-0.03	-0.03
**Omnivores**	0.01	0.00		0.02	0.03
**Plants (Other)**	0.08	0.02	0.11		0.01
**Vertebrates. & Scavengers**	0.26	0.02	0.03	0.26	

(B)

**6** / **p_x**	**Fruit & Nectar**	**Invertebrates**	**Omnivores**	**Plants (Other)**	**Vertebrates. & Scavengers**
**Fruit & Nectar**		0.00	0.00	0.02	0.00
**Invertebrates**	0.48		0.02	0.02	0.02
**Omnivores**	0.48	0.00		0.00	-0.02
**Plants (Other)**	0.01	0.00	0.35		-0.02
**Vertebrates. & Scavengers**	0.36	0.00	0.01	0.01	

Supplementary References

1 García-Peña, G. E., Sol, D., Iwaniuk, A. N. & Székely, T. Sexual selection on brain size in shorebirds (Charadriiformes). *Journal of Evolutionary Biology* **26**, 878-888 (2013). https://doi.org:10.1111/jeb.12104

2 Kempenaers, B. Mating systems in birds. *Current Biology* **32**, R1115-R1121 (2022).

3 Székely, T., Reynolds, J. D. & Figuerola, J. Sexual size dimorphism in shorebirds, gulls, and alcids: the influence of sexual and natural selection. *Evolution* **54**, 1404-1413 (2000).

4 Baker, J., Meade, A., Pagel, M. & Venditti, C. Positive phenotypic selection inferred from phylogenies. *Biological Journal of the Linnean Society* **118**, 95-115 (2016).

5 Venditti, C., Meade, A. & Pagel, M. Multiple routes to mammalian diversity. *Nature* **479**, 393-396 (2011). https://doi.org:10.1038/nature10516

6 Venditti, C., Baker, J. & Barton, R. A. Co-evolutionary dynamics of mammalian brain and body size. *Nature Ecology & Evolution* **8**, 1534-1542 (2024).

7 Baker, J., Humphries, S., Ferguson-Gow, H., Meade, A. & Venditti, C. Rapid decreases in relative testes mass among monogamous birds but not in other vertebrates. *Ecology Letters* **n/a** (2019). https://doi.org:10.1111/ele.13431

8 Federhen, S. The NCBI taxonomy database. *Nucleic acids research* **40**, D136-D143 (2012).

9 Froese, R. & Pauly, D. *FishBase*, <http://www.fishbase.org> (2012).

10 Tsuboi, M. *et al.* Breakdown of brain–body allometry and the encephalization of birds and mammals. *Nature Ecology & Evolution* **2**, 1492-1500 (2018). https://doi.org:10.1038/s41559-018-0632-1

11 R: A language and environment for statistical computing (R Foundation for Statistical Computing, 2024).

12 Hadfield, J. D. MCMC methods for multi-response generalized linear mixed models: the MCMCglmm R package. *Journal of Statistical Software* **33**, 1-22 (2010). https://doi.org:10.18637/jss.v033.i02

13 van de Pol, M. & Wright, J. A simple method for distinguishing within-versus between-subject effects using mixed models. *Animal Behaviour* **77**, 753-758 (2009). https://doi.org:10.1016/j.anbehav.2008.11.006

14 Isler, K. & Van Schaik, C. P. Metabolic costs of brain size evolution. *Biology letters* **2**, 557-560 (2006).

15 Kverková, K. *et al.* The evolution of brain neuron numbers in amniotes. *Proceedings of the National Academy of Sciences* **119**, e2121624119 (2022).

16 Beaulieu-Laroche, L. *et al.* Allometric rules for mammalian cortical layer 5 neuron biophysics. *Nature* **600**, 274-278 (2021).

17 DeCasien, A. R., Williams, S. A. & Higham, J. P. Primate brain size is predicted by diet but not sociality. *Nature ecology & evolution* **1**, 0112 (2017).

18 Gillooly, J. F. & McCoy, M. W. Brain size varies with temperature in vertebrates. *PeerJ* **2**, e301 (2014).

19 Wilson, L. N. *et al.* Global latitudinal gradients and the evolution of body size in dinosaurs and mammals. *Nat Comm* **15**, 2864 (2024). https://doi.org:10.1038/s41467-024-46843-2

20 Wilman, H. *et al.* EltonTraits 1.0: Species-level foraging attributes of the world's birds and mammals. *Ecology* **95**, 2027-2027 (2014).
