# Supplementary material for: Macroevolutionary brain scaling is a microevolutionary metaphenomenon
Source: Nat Commun. 2025 Dec 4;17:136. doi: 10.1038/s41467-025-66843-0 (PMC12775500; doi:10.1038/s41467-025-66843-0)
Supplement: Supplementary file 2 — Descriptions of Additional Supplementary Files [file 41467_2025_66843_MOESM2_ESM.pdf]

Description of Additional Supplementary Files

Supplementary Data 1. (separate file)
Dataset used in our main analysis, along with all sources used. This dataset contains the following columns:

- *Species: The name of the taxon for which data was obtained, linked to the TimeTree*
- *Class: Major animal group to which this taxon belongs.*
- *Subclade: Subclade used in our Subclade analyses.*
- *Sub-subclade: For groups showing deviations, further divisions in sub-clade based on data and phylogeny (see Supplementary Note 1 and 5).*
- *Bd_g: body size, in grams*
- *Br_g: brain size, in grams*
- *Source: The original reference from which this data was obtained.*

Supplementary Data 2. (separate file)
Branch-specific rate estimates. This tab-delimited text file contains the following columns:

- *Descendant taxa: which taxa does this branch lead to (a terminal branch will have only a single species; a root branch would lead to all species)*
- *Number of descendants: How many taxa define this branch?*
- *Median Scalar: What is the median rate parameter estimated along this branch?*
- *Class: Which class or classes does this branch lead to (or fall within)*
- *Sub-clade: Which sub-clade(s) does this branch lead to (or fall within)*
- *Percentage scaled: In what proportion of the posterior distribution is a rate scalar estimated to fall along this branch?*
- *Phenotypic selection: Does this branch conform to the criteria for phenotypic selection (>2 in >95% of the posterior is positive phenotypic selection; <0.5 in >95% of the posterior is negative phenotypic selection).*

Supplementary Code 1. (separate file)
Custom R function used to identify and delete tips in the tree which share zero-length terminal branches.
