# Supplementary material for: Macroevolutionary brain scaling is a microevolutionary metaphenomenon
Source: Nat Commun. 2025 Dec 4;17:136. doi: 10.1038/s41467-025-66843-0 (PMC12775500; doi:10.1038/s41467-025-66843-0)
Supplement: Supplementary file 6 — Reporting Summary [file 41467_2025_66843_MOESM6_ESM.pdf]

nature portfolio

Corresponding author(s): Joanna Baker and Chris Venditti

Last updated by author(s): 31/10/2025

Reporting Summary

Nature Portfolio wishes to improve the reproducibility of the work that we publish. This form provides structure for consistency and transparency in reporting. For further information on Nature Portfolio policies, see our Editorial Policies and the Editorial Policy Checklist.

Statistics

For all statistical analyses, confirm that the following items are present in the figure legend, table legend, main text, or Methods section.

n/a	Confirmed	
☐	☒	The exact sample size (*n*) for each experimental group/condition, given as a discrete number and unit of measurement
☒	☐	A statement on whether measurements were taken from distinct samples or whether the same sample was measured repeatedly
☐	☒	The statistical test(s) used AND whether they are one- or two-sided<br>*Only common tests should be described solely by name; describe more complex techniques in the Methods section.*
☐	☒	A description of all covariates tested
☐	☒	A description of any assumptions or corrections, such as tests of normality and adjustment for multiple comparisons
☐	☒	A full description of the statistical parameters including central tendency (e.g. means) or other basic estimates (e.g. regression coefficient) AND variation (e.g. standard deviation) or associated estimates of uncertainty (e.g. confidence intervals)
☐	☒	For null hypothesis testing, the test statistic (e.g. *F*, *t*, *r*) with confidence intervals, effect sizes, degrees of freedom and *P* value noted<br>*Give P values as exact values whenever suitable.*
☐	☒	For Bayesian analysis, information on the choice of priors and Markov chain Monte Carlo settings
☐	☒	For hierarchical and complex designs, identification of the appropriate level for tests and full reporting of outcomes
☒	☐	Estimates of effect sizes (e.g. Cohen's *d*, Pearson's *r*), indicating how they were calculated

Our web collection on statistics for biologists contains articles on many of the points above.

Software and code

Policy information about availability of computer code

Data collection	We did not use any custom code or software for data collection.
Data analysis	We used a custom R function in our analysis, which we provide as supplementary material. Our main analyses are conducted using BayesTraitsV5.

For manuscripts utilizing custom algorithms or software that are central to the research but not yet described in published literature, software must be made available to editors and reviewers. We strongly encourage code deposition in a community repository (e.g. GitHub). See the Nature Portfolio guidelines for submitting code & software for further information.

Data

Policy information about availability of data

All manuscripts must include a data availability statement. This statement should provide the following information, where applicable:

- Accession codes, unique identifiers, or web links for publicly available datasets
- A description of any restrictions on data availability
- For clinical datasets or third party data, please ensure that the statement adheres to our policy

Data availability
All data analysed in this study are available in the main text or the supplementary materials, along with the original sources from which they were obtained (Supplementary Data 1). Source data are provided as a Source Data file.

Code availability
We provide code developed as a part of this work in the supplementary material (Supplementary Code 1), but all other analyses were performed using publicly available, published and peer-reviewed programs which are cited appropriately in the text.

Research involving human participants, their data, or biological material

Policy information about studies with human participants or human data. See also policy information about sex, gender (identity/presentation), and sexual orientation and race, ethnicity and racism.

Reporting on sex and gender	Does not apply for our study.
Reporting on race, ethnicity, or other socially relevant groupings	Does not apply for our study.
Population characteristics	Does not apply for our study.
Recruitment	Does not apply for our study.
Ethics oversight	Does not apply for our study.

Note that full information on the approval of the study protocol must also be provided in the manuscript.

Field-specific reporting

Please select the one below that is the best fit for your research. If you are not sure, read the appropriate sections before making your selection.

☐ Life sciences ☐ Behavioural & social sciences ☒ Ecological, evolutionary & environmental sciences

For a reference copy of the document with all sections, see nature.com/documents/nr-reporting-summary-flat.pdf

Ecological, evolutionary & environmental sciences study design

All studies must disclose on these points even when the disclosure is negative.

Study description	We studied the relationship between brain and body size across animals using phylogenetic regression models.
Research sample	Species-level brain and body size data for 4679 animals. Individual-level brain and body size data for 376 vertebrate species.
Sampling strategy	We used all available data from previously published literature. Species-level averages were used.
Data collection	Data was was collected and checked by JB. All data was obtained from the literature and was not measured as a part of this study.
Timing and spatial scale	Data was collected from public data bases and published literature. Details are available in the Methods section.
Data exclusions	Three species were excluded from the analysis owing to extreme values likely attributable to error: Arctocephalus tropicalis, Malacothrix typicus, and Balaenoptera musculus. We describe these in more detail in our supplementary information and methods.
Reproducibility	All analyses were conducted multiple times to ensure repeatability.
Randomization	This is not relevant to our study because it is not experimental in this way.
Blinding	Blinding is not relevant to our study as it is not experimental in this way.

Did the study involve field work? ☐ Yes ☒ No

Reporting for specific materials, systems and methods

We require information from authors about some types of materials, experimental systems and methods used in many studies. Here, indicate whether each material, system or method listed is relevant to your study. If you are not sure if a list item applies to your research, read the appropriate section before selecting a response.

Materials & experimental systems

n/a	Involved in the study	
☒	☐	Antibodies
☒	☐	Eukaryotic cell lines
☒	☐	Palaeontology and archaeology
☒	☐	Animals and other organisms
☒	☐	Clinical data
☒	☐	Dual use research of concern
☒	☐	Plants

Methods

n/a	Involved in the study	
☒	☐	ChIP-seq
☒	☐	Flow cytometry
☒	☐	MRI-based neuroimaging

Plants

Seed stocks	Does not apply for our study.
Novel plant genotypes	Does not apply for our study.
Authentication	Does not apply for our study.
